# Genetically Predicted Association of 91 Circulating Inflammatory Proteins with Multiple Sclerosis: A Mendelian Randomization Study

**DOI:** 10.3390/brainsci14080833

**Published:** 2024-08-19

**Authors:** Xin’ai Li, Zhiguo Ding, Shuo Qi, Peng Wang, Junhui Wang, Jingwei Zhou

**Affiliations:** 1 Department of Thyropathy, Dongzhimen Hospital, Beijing University of Chinese Medicine, Beijing 100013, China; xinaili@bucm.edu.cn (X.L.); dingzhiguo_1@163.com (Z.D.); shuoqi@bucm.edu.cn (S.Q.); 2 Sun Simiao Institute, Beijing University of Chinese Medicine, Tongchuan 727000, China; 3 Thyropathy Hospital, Sun Simiao Hospital, Beijing University of Chinese Medicine, Tongchuan 727000, China; 4 The Key Laboratory of Cardiovascular Remodelling and Function Research, Chinese Ministry of Education and Chinese Ministry of Public Health, Department of Cardiology, Qilu Hospital of Shandong University, Jinan 250012, China; 17866806032@163.com; 5 Lunenfeld-Tanenbaum Research Institute, Mount Sinai Hospital, Toronto, ON M5G 1X5, Canada; 6 The 1st Ward, Department of Nephrology and Endocrinology, Dongzhimen Hospital, Beijing University of Chinese Medicine, Beijing 100010, China

**Keywords:** multiple sclerosis, circulating inflammatory protein, mendelian randomization, biomarkers

## Abstract

Previous studies have validated a close association between inflammatory factors and multiple sclerosis (MS), but their causal relationship is not fully profiled yet. This study used Mendelian randomization (MR) to investigate the causal effect of circulating inflammatory proteins on MS. Data from a large-scale genome-wide association study (GWAS) were analyzed using a two-sample MR method to explore the relationship between 91 circulating inflammatory proteins and MS. The inverse-variance-weighted (IVW) analysis was employed as the main method for evaluating exposures and outcomes. Furthermore, series of the methods of MR Egger, weighted median, simple mode, and weighted mode were used to fortify the final results. The results of the IVW method were corrected with Bonferroni (bon) and false discovery rate (fdr) for validating the robustness of results and ensuring the absence of heterogeneity and horizontal pleiotropy. The sensitivity analysis was also performed. The results of the forward MR analysis showed that higher levels of CCL25 were found to be associated with an increased risk of MS according to IVW results, OR: 1.085, 95% CI (1.011, 1.165), *p* = 2.42 × 10^−2^, adjusted p_adj_bon = 1, p_adj_fdr = 0.307. Similarly, higher levels of CXCL10 were found to be associated with an increased risk of MS, OR: 1.231, 95% CI (1.057, 1.433), *p* = 7.49 × 10^−3^, adjusted p_adj_bon = 0.682, p_adj_fdr = 0.227. In contrast, elevated levels of neurturin (NRTN) were associated with a decreased risk of MS, OR: 0.815, 95% CI (0.689, 0.964), *p* = 1.68 × 10^−2^, adjusted p_adj_bon = 1, p_adj_fdr = 0.307. Reverse MR analysis showed no causal relationship between MS and the identified circulating inflammatory cytokines. The effects of heterogeneity and level pleiotropy were further excluded by sensitivity analysis. This study provides new insights into the relationship between circulating inflammatory proteins and MS and brings up a new possibility of using these cytokines as potential biomarkers and therapeutic targets. The data in this study show that there are only weak associations between inflammatory molecules and MS risk, which did not survive bon and fdr correction, and the obtained *p*-values are quite low. Therefore, further studies on larger samples are needed.

## 1. Introduction

Characterized by reactive gliosis, axonal damage, neuronal degeneration, and inflammatory cell infiltration, MS is an autoimmune disorder affecting the central nervous system (CNS) [1]. The occurrence of this disease is mainly associated with genetic factors [2], environmental factors [3], lifestyle [4], viral infection [5], and immunologic factors [6]. Its pathology is featured by a loss of myelin sheath in the CNS with infiltration of a large number of inflammatory cells [7]. It leads to a heterogeneous set of symptoms and signs due to involvement of different motor, sensory, and autonomic nervous systems [8]. Patients usually present with neurological symptoms such as cognitive impairment, motor ataxia, blindness, and loss of coordination [9]. The disease poses a significant burden to patients and families [10]. The prevalence of MS has been increasing globally [11]. Therefore, identifying modifiable risk factors is an imperative task of the medical community for the purpose of developing novel strategies to manage the disease.

Chronic inflammatory response in MS arises from the activation of innate and adaptive immune responses in the CNS [12]. Although the CNS is considered an immune-privileged organ with highly controlled adaptive immunity and inflammation [13], recent findings have shed light on the fact that neuroinflammation or neuroimmune response play an essential role on the development of neurodegenerative conditions [14,15]. Among these, several cytokines with elevated expression levels in MS patients have been considered to be important biomarkers for this disease [16,17]. However, comprehensive preclinical and clinical studies are still not in place, and the clinical significance of these cytokines is not yet fully understood. The study of identifying certain circulating inflammatory proteins that are closely associated with MS may provide a new perspective in terms of the diagnosis and treatment of MS.

Genetic variation is the foundation for causal inference in MR. In order to deduce the influence of biological factors on disease, the fundamental idea involves utilizing the influence of randomly assigned genotypes on phenotype [18,19]. This method is effective in diminishing the effects of biases and confounders caused by behavioral or environmental influences while relying on the random distribution of genetic variation during meiosis [20]. When dealing with rare diseases, it proves to be highly effective in tackling the drawbacks of conventional randomized controlled trials and observational studies [21]. The GWAS database was utilized for data mining in order to facilitate a two-sample MR analysis, which aimed to uncover the causal association between circulating inflammatory proteins and MS. Our primary aim is to investigate how genetic proxy inflammatory protein levels influence the likelihood of developing MS.

## 2. Methods

### 2.1. Study Design

**Figure 1:**
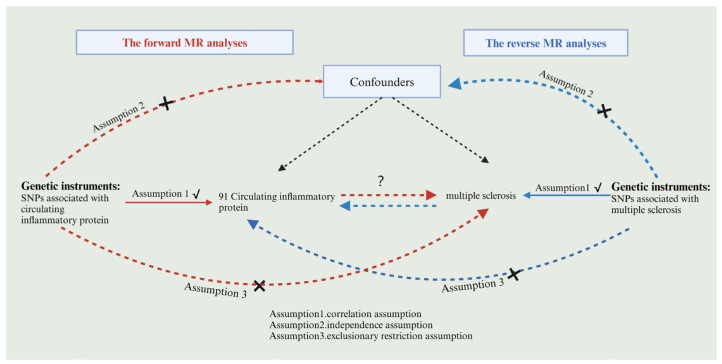
Flowchart of bidirectional MR analysis. Red arrows indicate the flowchart for forward MR and blue arrows indicate the flowchart for reverse MR, black arrows indicate forward MR and reverse MR sharing.

**Figure 2:**
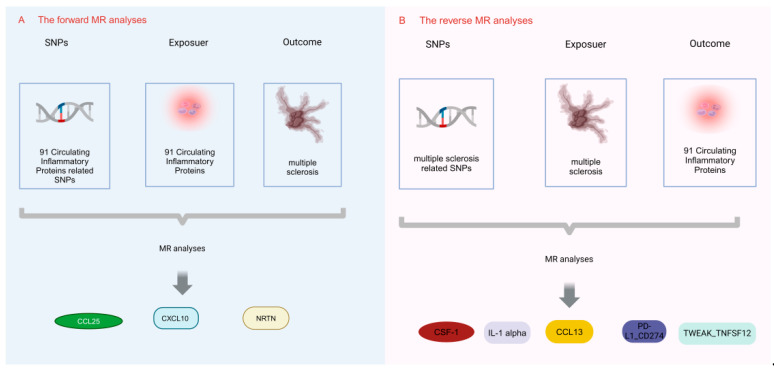
Visual summary of the analysis. The MR analysis unveils the relationships between circulating inflammatory proteins and MS. (**A**) The forward MR analyses; (**B**) the reverse MR analyses.

Using the two-sample MR study, this research was performed to examine the causal link between circulating inflammatory proteins and MS. Achieving valid results in MR analysis hinges on satisfying three key assumptions. Single nucleotide polymorphisms (SNPs) were used as instrumental variables (IVs) in this study, and it is vital for IVs acting as risk factors to meet three conditions, as illustrated in Figure 1. First, a reliable connection to the risk factor being evaluated must be established (correlation assumption). Second, it is essential to ensure independence from any recognized or unrecognized confounding variables (independence assumption). Third, only the risk factor should influence the outcome, while any other direct causal pathway will be excluded (exclusionary restriction assumption) [20]. Through the utilization of openly accessible data derived from extensive GWAS and consortia, ethical clearance was not a prerequisite for the conduct of this study. Visual summary of the analysis is shown in Figure 2.

### 2.2. Selection of IVs for MR Analyses

Genetic variants achieving genome-wide significance were recognized as IVs. We adjusted the criteria by increasing the threshold to *p* < 5 × 10^−6^ to incorporate more inflammatory proteins for all 91 inflammatory proteins, and this allowed us the opportunity to retrieve IVs. Similarly, in the reverse analysis, SNPs that reached the threshold *p* < 5 × 10^−6^ were used as IVs for MS. To maintain variant independence, SNPs with high linkage disequilibrium (specified as r^2^ > 0.001 and kb < 10,000 kb) were omitted. Variants with conflicting allelic frequencies underwent harmonization or elimination to align with the estimated effects. Palindromic SNPs were adjusted based on a maximum minor allele frequency (MAF) criterion set at 0.01. In instances where the results lacked directly related SNPs for the exposure, we opted for proxy SNPs exhibiting high correlation (r^2^ > 0.8) with the desired variant. These rigorously selected SNPs served as the definitive genetic IVs for the ensuing MR evaluations. For each SNP, the F-statistic was derived using Beta^2^/se^2^, where Beta symbolizes the estimated effect allele for the exposure and SE indicated the standard error. The formula 2 × Beta^2^ × MAF × (1 − MAF) [22] was employed to compute the variance fraction attributed to each SNP. An F-statistic exceeding 10 indicated a robust correlation between the IV and exposure, ensuring that the MR outcomes remained unaffected by weak instrument bias [23].

### 2.3. Data Sources

We obtained summary data related to MS from GWAS, including genetic data from 47,429 cases and 68,374 subjects from controls of European ancestry [24]. In 11 cohorts, 91 circulating inflammatory proteins were identified from a population of 14,824 individuals of European origin. The original paper describes the procedures utilized for assessing inflammatory proteins [25]. The complete per-protein GWAS summary statistics can be downloaded at https://www.phpc.cam.ac.uk/ceu/proteins (accessed on 1 June 2024) and from the EBI GWAS catalog (accession number GCST90274758-GCST90274848). Between the exposure and outcome groups, specific information on the 91 circulating inflammatory proteins is shown in Table 1. There will be no overlap in population selection.

### 2.4. MR and Sensitivity Analysis

The results of five Mendelian methods, MR Egger [26], weighted median [27], IVW [28,29], simple mode, and weighted mode [30], were used, and the results of the IVW method were corrected with bon and fdr [31]. When there were no multiple validities in IV [32], the IVW method demonstrated the greatest statistical validity and effectiveness [32]. Hence, IVW was employed as the primary research methodology in this study [33,34,35]. Furthermore, the techniques we incorporated, specifically weighted mode, simple mode, weighted median, and MR Egger, enhanced the conclusive findings [26,27]. By applying Cochran’s Q test, the heterogeneity of SNPs in IVW and MR Egger was evaluated, consequently bolstering the robustness of the results [36]. The intercept of MR-Egger [26] was deployed to examine horizontal pleiotropy [26]. In order to ascertain whether a single SNP was the sole factor influencing the causal effect, we conducted a leave-one-out analysis [37]. The detection of pleiotropic residuals and outliers was executed using MR-Presso [26]. MR-Steiger was employed to establish the correct direction of causality. In instances where the exposure was anticipated to result in the outcome, it was classified as TRUE; if not, it was marked as FALSE [38]. All analyses were two-sided and executed through the Two Sample MR and MRPRESSO packages in R software version 4.3.2.

## 3. Results

### 3.1. Effect of 91 Circulating Inflammatory Proteins on MS

In the forward MR analysis, details of the genetic tools used to assess the effects of 91 plasma proteins on MS were recorded separately (Table S1). All MS-associated SNPs used as IVs had F-statistics higher than 10, suggesting a strong prediction of MS, whereas there was less evidence of weak IV bias in our study. According to the IVW results, higher levels of CCL25 were found to be associated with an increased risk of MS, OR: 1.085, 95% CI (1.011, 1.165) *p* = 2.42 × 10^−2^, adjusted p_adj_bon = 1, p_adj_fdr = 0.307. Similarly, there was an association between higher levels of CXCL10 and increased risk of MS, OR: 1.231, 95% CI (1.057, 1.433), *p* = 7.49 × 10^−3^, adjusted p_adj_bon = 0.682, p_adj_fdr = 0.227. On the contrary, elevated NRTN levels were associated with a reduced risk of MS, OR: 0.815, 95% CI (0.689, 0.964), *p* = 1.68 × 10^−2^, adjusted p_adj_bon = 1, p_adj_fdr = 0.307, as shown in Table 2.

### 3.2. Effect of MS on 91 Circulating Inflammatory Proteins

In the inverse MR analysis, we found that MS was associated with five circulating inflammatory proteins, among which IL-1 alpha was positively causally associated with MS: *p* = 3.30 × 10^−4^, OR:1.035, 95% CI (1.016, 1.054), adjusted p_adj_bon = 0.026, p_adj_fdr = 0.026; and the blood inflammatory factor CSF-1: *p* = 2.35 × 10^−2^, OR: 0.983, 95% CI (0.969, 0.998), adjusted p_adj_bon = 1, p_adj_fdr = 0.376; CCL13: *p* = 1.45 × 10^−2^, OR: 0.980, 95% CI (0.964, 0.996), adjusted p_ adj_bon = 1, p_adj_fdr = 0.300; PD-L1_CD274: *p* = 1.50 × 10^−2^, OR:0.982, 95% CI (0.968, 0.996), adjusted p_adj_bon = 1, p_adj_fdr = 0.300; TWEAK_TNFSF12: *p* = 1.43 × 10^−2^, OR: 0.981, 95% CI (0.966, 0.996), adjusted p_adj_bon = 1, p_adj_fdr = 0.300 were negatively causally associated with MS, as shown in Table 3. However, no bi-directional genetic causality was found. Genetic tools used to assess the association of MS and 91 plasma proteins were documented in Table S2.

### 3.3. Sensitivity Analysis

**Figure 3:**
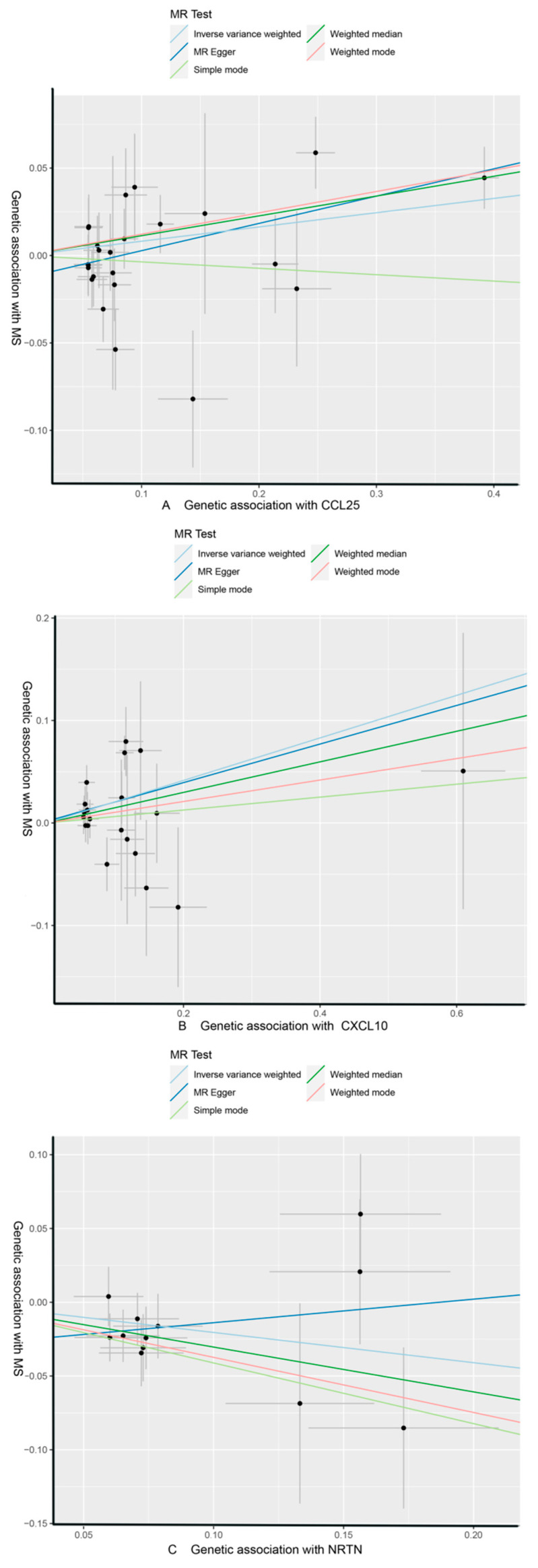
CCL25, CXCL10, and NRTN of MS with scatter plots, respectively. (**A**) MS as the outcome, with CCL25 as the exposure; (**B**) MS as the outcome, with CXCL10 as the exposure; (**C**) MS as the outcome, with NRTN as the exposure.

**Figure 4:**
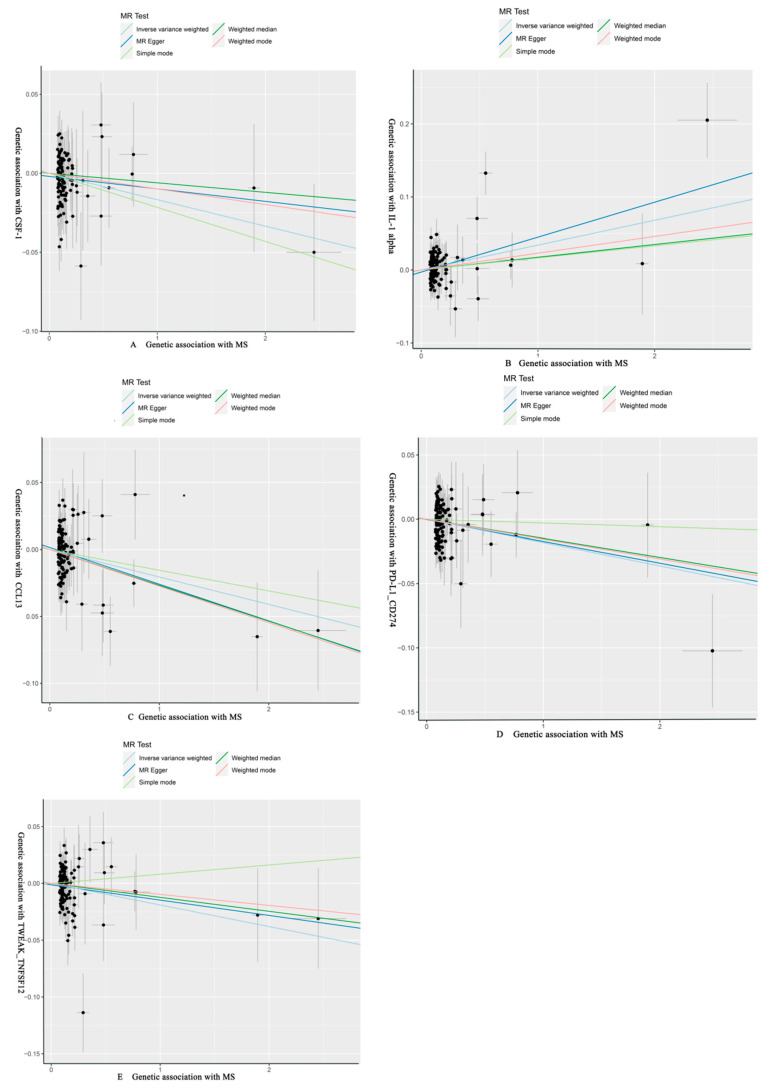
MS of CSF-1, IL-1 alpha, CCL13, PD-L1_CD274, TWEAK_TNFSF12 with scatter plots, respectively. (**A**) CSF-1 as the outcome, with MS as the exposure; (**B**) IL-1 alpha as the outcome, with MS as the exposure; (**C**) CCL13 as the outcome, with MS as the exposure; (**D**) PD-L1_CD274 as the outcome, with MS as the exposure; (**E**) TWEAK_TNFSF12 as the outcome, with MS as the exposure.

**Figure 5:**
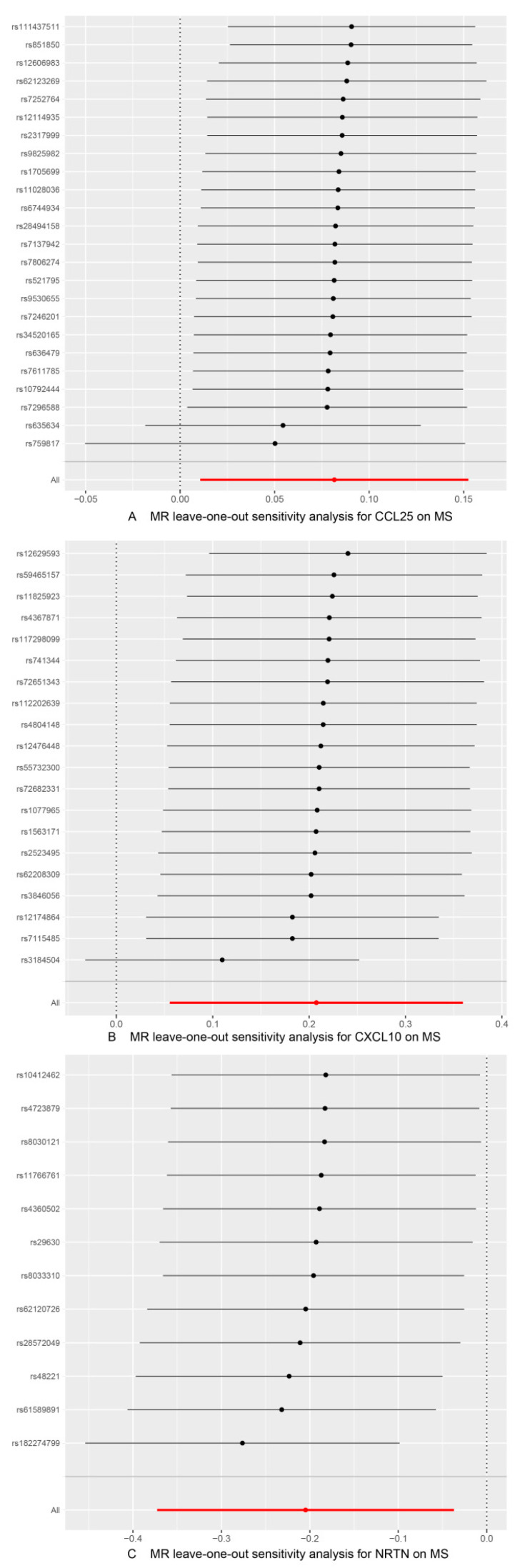
Use of the IVW method to display the results of leave-one-out analyses and assess the impact of individual SNPs on the overall MR results by excluding each SNP in turn. (**A**) MS as the outcome, with CCL25 as the exposure; (**B**) MS as the outcome, with CXCL10 as the exposure; (**C**) MS as the outcome, with NRTN as the exposure.

**Figure 6:**
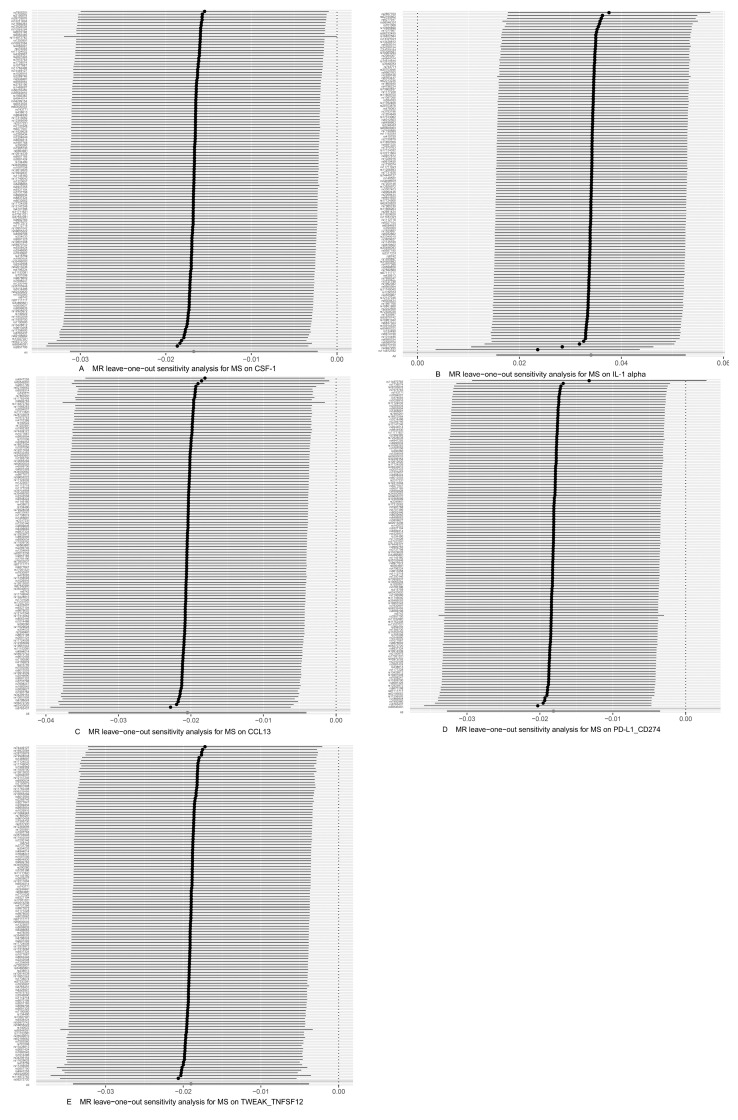
Use of the IVW method to display the results of leave-one-out analyses and assess the impact of individual SNPs on the overall MR results by excluding each SNP in turn. (**A**) CSF-1 as the outcome, with MS as the exposure; (**B**) IL-1 alpha as the outcome, with MS as the exposure; (**C**) CCL13 as the outcome, with MS as the exposure; (**D**) PD-L1_CD274 as the outcome, with MS as the exposure; (**E**) TWEAK_TNFSF12 as the outcome, with MS as the exposure.

As shown in Table 4 and Table 5, in the IVW and MR-Egger analysis based on Cochran’s Q test, the results indicated no heterogeneity of SNPs. No signs of horizontal pleiotropy were found in the MR-Egger intercept. The MR-Presso method did not identify any outliers. In addition, scatter plots ruled out potential outliers and horizontal pleiotropy (Figure 3 and Figure 4). In addition, no SNPs with large effect sizes were tested for bias estimation by the leave-one-out test (Figure 5 and Figure 6). The MR-Steiger analysis results validated the accuracy of the directionality and ruled out any indication of reverse causality. Sensitivity analysis eliminated the impacts of horizontal pleiotropy and heterogeneity, confirming the reliability of the outcomes. Presently, there is evidence from MS indicating a connection between MS and inflammatory proteins [39,40,41,42]. Nevertheless, the precise cause and effect association is still unclear at the genetic level as a result of constraints in research. In the context of the potential causal link of 91 circulating inflammatory proteins with MS in this exploratory study, we completed a comprehensive two-sample MR analysis. 

## 4. Discussion 

In this study, bidirectional MR analysis was performed to explore the association of 91 circulating inflammatory cytokine proteins with MS. The aim was to explore genetic evidence for a potential causal relationship between circulating inflammatory cytokines and MS risk. Our study showed that CCL25, CXCL10, and NRTN levels were associated with the likelihood of developing MS according to forward analysis. In addition, reverse MR analysis showed that CSF-1, IL-1 alpha, CCL13, PD-L1_CD274, and TWEAK_TNFSF12 were genetically causally associated with MS. No circulating inflammatory proteins were found to be bi-directionally causally associated with the disease. This study is the first to explore the interrelationship between inflammatory proteins and MS through bidirectional MR analysis. This study provides some evidence to use medications targeting inflammatory factors to treat MS in the future.

Focal cerebral white matter lesions characterized by inflammation and demyelination are the most obvious hallmark of MS histopathology. The inflammatory infiltrate consists mainly of phagocytes, T cells, and B cells originating from the blood [43]. Cortical lesions present in early MS are associated with significant inflammation [44]. It has been shown that Th1 and Th17 responses are the main cause of MS progression [45]. It has also been shown [46] that the neutrophil–lymphocyte ratio (NLR) is significantly increased in MS patients compared to controls. A study has shown that impairment of CD200-CD200R-mediated macrophage silencing exacerbates CNS inflammation and neuronal degeneration [47]. Our study showed that high levels of CCL25 and CXCL10 were associated and positively correlated with the development of MS, while NRTN levels were negatively correlated with MS risk. This suggests that elevated levels of CCL25 and CXCL10 could promote the development of MS, whereas elevated NRTN could reduce the risk of developing MS. A notable feature of this study was the use of MR analysis to assess the pathogenic impact of circulating inflammatory proteins on MS risk. An MR approach could skillfully handle confounding factors, reverse causality, and increase the confidence in causal inferences. These results could position these proteins as potential biomarkers for MS diagnosis and provide a new way to extensively understand the pathogenesis of the disease.

CCL25 is classified as a chemokine that is expressed in the thymus [48]. CCL25 is predominantly found in the intestinal epithelium and thymus. However, other parenchymal cells, such vascular endothelial cells, can produce it as well. These CCL25 expression cells can guide immature T cells to migrate into the thymus, where they turn mature and release [49], and subsequently are able to engage in numerous inflammatory responses. In recent years, research has brought more evidence of how CCR9/CCL25 contributes to inflammation, which are associated with several diseases, including cardiovascular disease (CVD), hepatitis, arthritis [50], inflammatory bowel disease [51], and asthma. Toll-like receptor 4 (TLR4) plays a role in the pathogenesis of experimental autoimmune encephalomyelitis (EAE) by regulating CCL25/CCR9 expression in response to Th17 infiltration [52]. Recent data suggest that CCR9 blockade or inhibition leads to a reduction in lymphocyte infiltration and amelioration of clinical symptoms in many clinical inflammatory disorders [53,54]. The fact that CCR9 mediates effector T-cell infiltration into the CNS suggests that CCL25/CCR9 is a potential new biologic target for the inhibiting of pathologic lymphocyte recruitment in MS therapy [55]. This indicates CCL25 as a risk factor for MS, which is revealed in this study as well (OR: 1.085, 95% CI: 1.011–1.165, *p* = 2.42 × 10^−2^, adjusted p_adj_bon = 1, p_adj_fdr = 0.307) and suggests its potential as a biomarker and therapeutic target of MS.

As a tiny protein, CXCL10 is an “inflammatory” chemokine that attaches to CXCR3 and enables immune response via leukocyte activation and recruitment, including eosinophils, T cells, NK cells, and monocytes [56]. Specimens of cerebrospinal fluid (CSF) were obtained from active MS patients and CXCL10 exhibited a higher level than those from the patients with non-inflammatory symptoms, according to the report of Sørensen et al. [57]. In these MS patients, CXCR3 was found to be expressed in over 90% of T cells from CSF, a substantially larger percentage than those T cells from peripheral blood. Previous studies have confirmed that the CXCL10/CXCR3 axis plays a critical role in MS patients [57,58,59]. In another study, Sørensen et al. [60] discovered that the CSF from MS patients contained significant CXCL10 levels, in line with the presence of more leukocytes. Furthermore, Comini-Frota et al. [61] discovered that the levels of serum CXCL10 were elevated among MS patients in comparison to the normal group. Here, our results also demonstrated that elevated CXCL10 levels were a risk factor of MS (OR: 1.231, 95% CI: 1.057–1.433, *p* = 7.49 × 10^−3^, adjusted p_adj_bon = 0.682, p_adj_fdr = 0.227), which is in line with the previous results.

There are a number of studies that have shown that administration of neurotrophic factors improves the survival of injured neurons in models of neuronal injury [62,63]. Neurotrophic factors show promise in promoting functional recovery following demyelination or nerve injury, making them good candidates in the study of MS to unveil pathogenesis and explore new treatment. Transplanted fibroblasts expressing BDNF or NT3 in adult rats with spinal cord injury could lead to improved myelin formation, OPC (oligodendrocyte progenitor cell) proliferation, and axonal growth [64]. When Schwann cells expressing BDNF or NT3 were transplanted into the spinal cord of demyelinated mice, they showed similar recovery of motor function [65]. The neuroglial cell-lineage-derived neurotrophic factor (GDNF) families, including NRTN, have been reported to play key roles in the maturation of neuromuscular synapses during development and post-nerve injury regeneration [66]. The results of this study suggest that NRTN has a potential protective effect on MS (OR: 0.815, 95% CI: 0.689–0.964, 1.68 × 10^−2^, adjusted p_adj_bon = 1, p_adj_fdr = 0.307), which is consistent with the above-mentioned results from the research of GDNF families. However, the underlying mechanism by which NRTN influences MS needs to be further investigated in future studies. 

This study employed MR analysis to evaluate the causal relationship between circulating inflammatory proteins and MS. This approach was selected to minimize confounding factors and potential reverse causation in causal inference. Genetic variants associated with these proteins were derived from recent GWAS meta-analyses, ensuring robust instrumental strength in MR analysis. The regression intercept tests of MR-PRESSO and MR-Egger were completed to determine multiplicity levels. To reduce the potential for bias, we applied a two-sample MR framework with outcome pooled data and exposure that does not overlap.

However, this study is subject to a few limitations. First, the exclusion of horizontal pleiotropy and IV assumptions were the specific assumptions integrated by the MR analysis. Sensitivity analyses were conducted to tackle these concerns. However, we cannot completely eliminate the possibility of unmeasured pleiotropy or confounders. Second, our research only involved individuals of European descent, potentially restricting the applicability of our conclusions to other demographic groups. Third, the obtained *p*-values are quite low, and no causal connection of circulating inflammatory proteins with MS had statistical significance in the wake of applying bon correction and fdr correction. The stringent parameters utilized in our analysis may have contributed to the false negative outcomes. Further studies on larger samples to confirm these findings are needed.

## 5. Conclusions

This study evaluated the potential causal relationship between 91 circulating inflammatory proteins and the risk of MS. We identified the plasma proteins CCL25 and CCL10 as being associated with an increased risk of MS, whereas NRTN was associated with a reduction in MS risk. However, only weak associations of inflammatory molecules and MS risk were found in our data, which did not survive bon and fdr correction. Therefore, further studies on larger samples are needed. The findings highlight that these inflammatory proteins in circulation are closely associated with MS to a certain extent, although they may not be the direct cause of MS. More research is needed to further substantiate these findings and investigate additional possible mechanisms for the association between inflammatory proteins and the risk of MS. However, the implications of these results are still significant for future studies in providing a research direction of deciphering the involvement of inflammation in MS and could help the development of new therapies of MS by targeting specific inflammatory pathways.

## Figures and Tables

**Table 1:** Ninety-one circulating inflammatory proteins.

Number	Abbreviations	Full Name	ID
1	CXCL9	Chemokine (C-X-C motif) ligand 9	GCST90274784
2	TWEAK_TNFSF12	Tumor Necrosis Factor-like Weak Inducer of Apoptosis—Tumor Necrosis Factor Superfamily Member 12	GCST90274846
3	CCL23	Chemokine (C-C motif) ligand 23	GCST90274767
4	ADA	Adenosine Deaminase	GCST90274759
5	CASP-8	Caspase-8	GCST90274763
6	CXCL6	Chemokine (C-X-C motif) ligand 6	GCST90274783
7	PD-L1_CD274	Programmed Cell Death 1 Ligand 1-Cluster of Differentiation 274	GCST90274832
8	IL-15RA	Interleukin-15 Receptor Alpha	GCST90274800
9	IL-1 alpha	Interleukin-1 alpha	GCST90274805
10	CST5	Cystatin 5	GCST90274777
11	IL-10	Interleukin-10	GCST90274795
12	IL-10RA	Interleukin-10 Receptor Alpha	GCST90274796
13	NTF3	Neurotrophin 3	GCST90274829
14	IL-12B	Interleukin-12B	GCST90274798
15	IL-33	Interleukin-33	GCST90274812
16	CCL13	Chemokine (C-C motif) ligand 13	GCST90274824
17	OPG_TNFRSF11B	Osteoprotegerin—Tumor Necrosis Factor Receptor Superfamily Member 11B	GCST90274830
18	IL-4	Interleukin-4	GCST90274813
19	LIF	Leukemia Inhibitory Factor	GCST90274819
20	FIt3L	Fms-related tyrosine kinase 3 ligand	GCST90274791
21	TNFRSF9	Tumor Necrosis Factor Receptor Superfamily Member 9	GCST90274841
22	IL-5	Interleukin-5	GCST90274814
23	DNER	Delta/Notch-like EGF repeat-containing receptor	GCST90274785
24	CCL20	Chemokine (C-C motif) ligand 20	GCST90274766
25	TNFSF14	Tumor Necrosis Factor Superfamily Member 14	GCST90274842
26	IL-6	Interleukin-6	GCST90274815
27	CCL19	Chemokine (C-C motif) ligand 19	GCST90274765
28	TNFB_LTA	Tumor Necrosis Factor B/Lymphotoxin-alpha	GCST90274840
29	SIRT2	Sirtuin 2	GCST90274834
30	STAMPB	Six transmembrane proteins of prostate B	GCST90274837
31	4EBP1_EIF4EBP1	Eukaryotic Translation Initiation Factor 4E-Binding Protein 1	GCST90274758
32	IL-18R1	Interleukin-18 Receptor 1	GCST90274804
33	LAP TGF-beta-1	Latency Associated Peptide Transforming Growth Factor-beta 1	GCST90274818
34	IL-7	Interleukin-7	GCST90274816
35	CXCL5	Chemokine (C-X-C motif) ligand 5	GCST90274782
36	NRTN	Neurturin	GCST90274828
37	IL-13	Interleukin-13	GCST90274799
38	CDCP1	CUB Domain Containing Protein 1	GCST90274775
39	TGF-alpha	Transforming Growth Factor-alpha	GCST90274838
40	FGF-21	Fibroblast Growth Factor 21	GCST90274788
41	SLAMF1	Signaling Lymphocytic Activation Molecule Family Member 1	GCST90274835
42	CXCL1	Chemokine (C-X-C motif) ligand 1	GCST90274779
43	TRAIL	Tumor Necrosis Factor-related Apoptosis-inducing Ligand	GCST90274843
44	IL-17C	Interleukin-17C	GCST90274802
45	MMP-1	Matrix Metalloproteinase-1	GCST90274826
46	CXCL11	Chemokine (C-X-C motif) ligand 11	GCST90274781
47	FGF-23	Fibroblast Growth Factor 23	GCST90274789
48	uPA_PLAU	Urokinase- type Plasminogen Activator (uPA)/Plasminogen Activator, Urokinase (PLAU)	GCST90274847
49	FGF-19	Fibroblast Growth Factor 19	GCST90274787
50	CX3CL1	Chemokine (C-X3-C motif) ligand 1	GCST90274778
51	CXCL10	Chemokine (C-X-C motif) ligand 10	GCST90274780
52	FGF-5	Fibroblast Growth Factor 5	GCST90274790
53	CCL25	Chemokine (C-C motif) ligand 25	GCST90274768
54	ARTN	Artemin	GCST90274760
55	VEGF_A	Vascular Endothelial Growth Factor A	GCST90274848
56	SCF_KITLG	Stem Cell Factor (SCF)/KIT Ligand (KITLG)	GCST90274833
57	ST1A1_SULT1A1	Sulfotransferase Family 1A Member 1 (SULT1A1)	GCST90274836
58	CD244	Cluster of Differentiation 244	GCST90274771
59	CCL11	Chemokine (C-C motif) ligand 11	GCST90274764
60	MMP-10	Matrix Metalloproteinase-10	GCST90274827
61	TRANCE	Tumor Necrosis Factor (TNF)-related Activation-induced Cytokine	GCST90274844
62	IL-2	Interleukin-2	GCST90274806
63	Beta-NGF_NGF	Beta-Nerve Growth Factor (NGF)	GCST90274762
64	IL-2RB	Interleukin-2 Receptor Subunit Beta	GCST90274811
65	OSM	Oncostatin M	GCST90274831
66	AXIN1	Axis Inhibition Protein 1	GCST90274761
67	CCL2	Chemokine (C-C motif) ligand 2	GCST90274821
68	CCL8	Chemokine (C-C motif) ligand 8	GCST90274822
69	CD6	Cluster of Differentiation 6	GCST90274774
70	MIP-1 alpha_CCL3	Macrophage Inflammatory Protein-1 alpha (MIP-1 alpha)/Chemokine (C-C motif) ligand 3 (CCL3)	GCST90274825
71	hGDNF_GDNF	Human Glial Cell Line-Derived Neurotrophic Factor (GDNF)	GCST90274792
72	CCL7	Chemokine (C-C motif) ligand 7	GCST90274823
73	LIF-R	Leukemia Inhibitory Factor Receptor	GCST90274820
74	EN-RAGE_S100A12	Extracellular Newly Identified RAGE Binding Protein (EN-RAGE)/S100 Calcium Binding Protein A12 (S100A12)	GCST90274786
75	CCL4	Chemokine (C-C motif) ligand 4	GCST90274770
76	HGF	Hepatocyte Growth Factor	GCST90274793
77	IL-17A	Interleukin-17A	GCST90274801
78	CD5	Cluster of Differentiation 5	GCST90274773
79	CSF-1	Colony-Stimulating Factor 1	GCST90274776
80	CCL28	Chemokine (C-C motif) ligand 28	GCST90274769
81	CD40	Cluster of Differentiation 40	GCST90274772
82	IL-22RA1	Interleukin-22 Receptor Subunit Alpha 1	GCST90274809
83	TNF	Tumor Necrosis Factor	GCST90274839
84	IL-18	Interleukin-18	GCST90274803
85	IL-20RA	Interleukin-20 Receptor Subunit Alpha	GCST90274808
86	IL-24	Interleukin-24	GCST90274810
87	IFNG	Interferon gamma	GCST90274794
88	TSLP	Thymic Stromal Lymphopoietin	GCST90274845
89	IL-20	Interleukin-20	GCST90274807
90	IL10RB	Interleukin 10 Receptor Subunit Beta	GCST90274797
91	IL-8	Interleukin-8	GCST90274817

**Table 2:** MR analysis of the causal association between circulating inflammatory proteins and risk of MS.

Exposure	Outcome	Method	Nsnp	B	Se	Pval	OR (95%CI)	P_adj_bon	P_adj_fdr
CCL25	MS	MR Egger	24	0.156	0.053	7.49 × 10^−3^	1.169 (1.054, 1.297)		
		Weighted median		0.113	0.044	9.72 × 10^−3^	1.120 (1.028, 1.220)		
		IVW		0.082	0.036	2.42 × 10^−2^	1.085 (1.011, 1.165)	1	0.307
		Simple mode		−0.037	0.100	7.17 × 10^−1^	0.964 (0.793, 1.172)		
		Weighted mode		0.122	0.039	5.17 × 10^−3^	1.130 (1.046, 1.221)		
CXCL10		MR Egger	20	0.188	0.188	3.30 × 10^−1^	1.207 (0.835, 1.746)		
		Weighted median		0.149	0.101	1.39 × 10^−1^	1.161 (0.953, 1.414)		
		IVW		0.208	0.078	7.49 × 10^−3^	1.231 (1.057, 1.433)	0.682	0.227
		Simple mode		0.063	0.181	7.31 × 10^−1^	1.065 (0.747, 1.518)		
		Weighted mode		0.105	0.215	6.32 × 10^−1^	1.110 (0.729, 1.692)		
NRTN		MR Egger	12	0.160	0.282	5.85 × 10^−1^	1.173 (0.674, 2.040)		
		Weighted median		−0.304	0.118	1.02 × 10^−2^	0.738 (0.585, 0.931)		
		IVW		−0.205	0.086	1.68 × 10^−2^	0.815 (0.689, 0.964)	1	0.307
		Simple mode		−0.411	0.187	5.00 × 10^−2^	0.663 (0.459, 0.956)		
		Weighted mode		−0.374	0.189	7.39 × 10^−2^	0.688 (0.475, 0.997)		

**Table 3:** MR analysis of the causal association between MS and risk of circulating inflammatory proteins.

Exposure	Outcome	Method	Nsnp	B	Se	Pval	OR (95%CI)	P_adj_bon	P_adj_fdr
MS	CSF-1	MR Egger	122	−0.008	0.011	4.75 × 10^−1^	0.992 (0.971, 1.014)		
		Weighted median		−0.006	0.012	6.06 × 10^−1^	0.994 (0.972, 1.017)		
		IVW		−0.017	0.007	2.35 × 10^−2^	0.983 (0.969, 0.998)	1	0.376
		Simple mode		−0.022	0.026	4.05 × 10^−1^	0.979 (0.930, 1.029)		
		Weighted mode		−0.010	0.011	3.60 × 10^−1^	0.990 (0.970, 1.011)		
	IL-1 alpha	MR Egger	122	0.048	0.015	1.46 × 10^−3^	1.049 (1.019, 1.080)		
		Weighted median		0.017	0.016	2.70 × 10^−1^	1.018 (0.987, 1.050)		
		IVW		0.034	0.009	3.30 × 10^−4^	1.035 (1.016, 1.054)	0.026	0.026
		Simple mode		0.017	0.0345	6.33 × 10^−1^	1.017 (0.950, 1.088)		
		Weighted mode		0.023	0.018	1.95 × 10^−1^	1.023 (0.989, 1.059)		
	CCL13	MR Egger	122	−0.027	0.013	3.10 × 10^−2^	0.973 (0.949, 0.997)		
		Weighted median		−0.027	0.012	3.12 × 10^−2^	0.974 (0.950, 0.998)		
		IVW		−0.020	0.008	1.45 × 10^−2^	0.980 (0.964, 0.996)	1	0.300
		Simple mode		−0.015	0.029	5.89 × 10^−1^	0.985 (0.932, 1.041)		
		Weighted mode		−0.027	0.011	1.81 × 10^−2^	0.973 (0.952, 0.995)		
	PD-L1_CD274	MR Egger	123	−0.017	0.0112	1.32 × 10^−1^	0.983 (0.962, 1.005)		
		Weighted median		−0.015	0.013	2.51 × 10^−1^	0.985 (0.961, 1.011)		
		IVW		−0.018	0.007	1.50 × 10^−2^	0.982 (0.968, 0.996)	1	0.300
		Simple mode		−0.003	0.026	9.10 × 10^−1^	0.997 (0.948, 1.049)		
		Weighted mode		−0.015	0.011	1.64 × 10^−1^	0.985 (0.964, 1.006)		
	TWEAK_TNFSF12	MR Egger	123	−0.0135	0.0116	2.49 × 10^−1^	0.987 (0.964, 1.009)		
		Weighted median		−0.0123	0.011	2.76 × 10^−1^	0.988 (0.9663, 1.010)		
		IVW		−0.019	0.008	1.43 × 10^−2^	0.981 (0.966, 0.996)	1	0.300
		Simple mode		0.008	0.027	7.68 × 10^−1^	1.008 (0.955, 1.064)		
		Weighted mode		−0.010	0.011	3.97 × 10^−1^	0.990 (0.969, 1.013)		

**Table 4:** Sensitive analysis of the causal association between circulating inflammatory proteins and risk of MS.

Inflammatory Proteins	Outcomes	SNPs	Cochran’s Q Test		MR-Egger Intercept		MR-Presso		MR-Steiger
			IVW	MR Egger	Egger Intercept	*p* Value	Global Test RSSobs	*p* Value	Causal Direction
CCL25	MS	24	0.165	0.273	−0.013	0.078	0.187	0.034 (Outlier-corrected, 0 Outlier)	TRUE
CXCL10		20	0.098	0.074	0.002	0.911	0.064	0.015 (Outlier-corrected, 0 Outlier)	TRUE
NRTN		12	0.501	0.581	−0.030	0.206	0.534	0.031 (Outlier-corrected, 0 Outlier)	TRUE

**Table 5:** Sensitive analysis of the causal association between MS and risk of circulating inflammatory proteins.

Exposure	Outcomes	Nsnp	Cochran’s Q Test		MR-Egger Intercept		MR-Presso		MR-Steiger
			IVW	MR Egger	Egger Intercept	*p* Value	Global Test RSSobs	*p* Value	Causal Direction
MS	CSF-1	122	0.59	0.595	−0.002	0.282	0.622	0.023 (Outlier-corrected, 0 Outlier)	TRUE
	IL-1 alpha	122	0.092	0.099	−0.003	0.222	0.081	0.0005 (Outlier-corrected, 0 Outlier)	TRUE
	CCL13	122	0.050	0.050	0.002	0.459	0.053	0.016 (Outlier-corrected, 0 Outlier)	TRUE
	PD-L1_CD274	123	0.639	0.615	−0.0003	0.885	0.616	0.014 (Outlier-corrected, 0 Outlier)	TRUE
	TWEAK_TNFSF12	123	0.269	0.257	−0.001	0.526	0.308	0.016 (Outlier-corrected, 0 Outlier)	TRUE

## Data Availability

Data are available in a publicly accessible repository. The original data used in the study are openly available in GWAS Catalog (ebi.ac.uk): https://www.ebi.ac.uk/gwas/ (accessed on 1 June 2024) and https://gwas.mrcieu.ac.uk/ (accessed on 1 June 2024).

## Supplementary Materials

The following supporting information can be downloaded from: https://www.mdpi.com/article/10.3390/brainsci14080833/s1. Table S1: Detailed information of genetic instruments of 91 plasma proteins for effect on multiple sclerosis. Table S2: Detailed information of genetic instruments of multiple sclerosis for effect on 91 plasma proteins.

## References

[1] Coclitu C., Constantinescu C.S., Tanasescu R. (2016). The future of multiple sclerosis treatments. Expert Rev. Neurother..

[2] Harirchian M.H., Fatehi F., Sarraf P., Honarvar N.M., Bitarafan S. (2018). Worldwide prevalence of familial multiple sclerosis: A systematic review and meta-analysis. Mult. Scler. Relat. Disord..

[3] Browne P., Chandraratna D., Angood C., Tremlett H., Baker C., Taylor B.V., Thompson A.J. (2014). Atlas of Multiple Sclerosis 2013: A growing global problem with widespread inequity. Neurology.

[4] Mandoj C., Renna R., Plantone D., Sperduti I., Cigliana G., Conti L., Koudriavtseva T. (2015). Anti-annexin antibodies, cholesterol levels and disability in multiple sclerosis. Neurosci. Lett..

[5] Voumvourakis K.I., Fragkou P.C., Kitsos D.K., Foska K., Chondrogianni M., Tsiodras S. (2022). Human herpesvirus 6 infection as a trigger of multiple sclerosis: An update of recent literature. BMC Neurol..

[6] Kamphuis W.W., Derada Troletti C., Reijerkerk A., Romero I.A., de Vries H.E. (2015). The blood-brain barrier in multiple sclerosis: microRNAs as key regulators. CNS Neurol. Disord. Drug Targets.

[7] Garg N., Smith T.W. (2015). An update on immunopathogenesis, diagnosis, and treatment of multiple sclerosis. Brain Behav..

[8] Ward M., Goldman M.D. (2022). Epidemiology and Pathophysiology of Multiple Sclerosis. Continuum.

[9] Dutra R.C., Moreira E.L., Alberti T.B., Marcon R., Prediger R.D., Calixto J.B. (2013). Spatial reference memory deficits precede motor dysfunction in an experimental autoimmune encephalomyelitis model: The role of kallikrein-kinin system. Brain Behav. Immun..

[10] GBD 2016 Multiple Sclerosis Collaborators (2019). Global, regional, and national burden of multiple sclerosis 1990–2016: A systematic analysis for the Global Burden of Disease Study 2016. Lancet Neurol..

[11] Walton C., King R., Rechtman L., Kaye W., Leray E., Marrie R.A., Robertson N., La Rocca N., Uitdehaag B., van der Mei I. (2020). Rising prevalence of multiple sclerosis worldwide: Insights from the Atlas of MS, third edition. Mult. Scler..

[12] Sun Y., Yu H., Guan Y. (2023). Glia Connect Inflammation and Neurodegeneration in Multiple Sclerosis. Neurosci. Bull..

[13] Rauf A., Badoni H., Abu-Izneid T., Olatunde A., Rahman M.M., Painuli S., Semwal P., Wilairatana P., Mubarak M.S. (2022). Neuroinflammatory Markers: Key Indicators in the Pathology of Neurodegenerative Diseases. Molecules.

[14] Schain M., Kreisl W.C. (2017). Neuroinflammation in Neurodegenerative Disorders-a Review. Curr. Neurol. Neurosci. Rep..

[15] Liu Z., Cheng X., Zhong S., Zhang X., Liu C., Liu F., Zhao C. (2020). Peripheral and Central Nervous System Immune Response Crosstalk in Amyotrophic Lateral Sclerosis. Front. Neurosci..

[16] Tehrani A.R., Gholipour S., Sharifi R., Yadegari S., Abbasi-Kolli M., Masoudian N. (2019). Plasma levels of CTRP-3, CTRP-9 and apelin in women with multiple sclerosis. J. Neuroimmunol..

[17] Grzegorski T., Iwanowski P., Kozubski W., Losy J. (2022). The alterations of cerebrospinal fluid TNF-alpha and TGF-beta2 levels in early relapsing-remitting multiple sclerosis. Immunol. Res..

[18] Evans D.M., Davey Smith G. (2015). Mendelian Randomization: New Applications in the Coming Age of Hypothesis-Free Causality. Annu. Rev. Genom. Hum. Genet..

[19] Davey Smith G., Hemani G. (2014). Mendelian randomization: Genetic anchors for causal inference in epidemiological studies. Hum. Mol. Genet..

[20] Emdin C.A., Khera A.V., Kathiresan S. (2017). Mendelian Randomization. Jama.

[21] Larsson S.C., Butterworth A.S., Burgess S. (2023). Mendelian randomization for cardiovascular diseases: Principles and applications. Eur. Heart J..

[22] Teumer A., Chaker L., Groeneweg S., Li Y., Di Munno C., Barbieri C., Schultheiss U.T., Traglia M., Ahluwalia T.S., Akiyama M. (2018). Genome-wide analyses identify a role for SLC17A4 and AADAT in thyroid hormone regulation. Nat. Commun..

[23] Shungin D., Winkler T.W., Croteau-Chonka D.C., Ferreira T., Locke A.E., Mägi R., Strawbridge R.J., Pers T.H., Fischer K., Justice A.E. (2015). New genetic loci link adipose and insulin biology to body fat distribution. Nature.

[24] International Multiple Sclerosis Genetics Consortium (2019). Multiple sclerosis genomic map implicates peripheral immune cells and microglia in susceptibility. Science.

[25] Zhao J.H., Stacey D., Eriksson N., Macdonald-Dunlop E., Hedman Å.K., Kalnapenkis A., Enroth S., Cozzetto D., Digby-Bell J., Marten J. (2023). Genetics of circulating inflammatory proteins identifies drivers of immune-mediated disease risk and therapeutic targets. Nat. Immunol..

[26] Bowden J., Davey Smith G., Burgess S. (2015). Mendelian randomization with invalid instruments: Effect estimation and bias detection through Egger regression. Int. J. Epidemiol..

[27] Bowden J., Davey Smith G., Haycock P.C., Burgess S. (2016). Consistent Estimation in Mendelian Randomization with Some Invalid Instruments Using a Weighted Median Estimator. Genet. Epidemiol..

[28] Burgess S., Scott R.A., Timpson N.J., Davey Smith G., Thompson S.G. (2015). Using published data in Mendelian randomization: A blueprint for efficient identification of causal risk factors. Eur. J. Epidemiol..

[29] Burgess S., Butterworth A., Thompson S.G. (2013). Mendelian randomization analysis with multiple genetic variants using summarized data. Genet. Epidemiol..

[30] Hartwig F.P., Davey Smith G., Bowden J. (2017). Robust inference in summary data Mendelian randomization via the zero modal pleiotropy assumption. Int. J. Epidemiol..

[31] Glickman M.E., Rao S.R., Schultz M.R. (2014). False discovery rate control is a recommended alternative to Bonferroni-type adjustments in health studies. J. Clin. Epidemiol..

[32] Burgess S., Davey Smith G., Davies N.M., Dudbridge F., Gill D., Glymour M.M., Hartwig F.P., Kutalik Z., Holmes M.V., Minelli C. (2019). Guidelines for performing Mendelian randomization investigations: Update for summer 2023. Wellcome Open Res..

[33] Burgess S., Dudbridge F., Thompson S.G. (2016). Combining information on multiple instrumental variables in Mendelian randomization: Comparison of allele score and summarized data methods. Stat. Med..

[34] Yavorska O.O., Burgess S. (2017). MendelianRandomization: An R package for performing Mendelian randomization analyses using summarized data. Int. J. Epidemiol..

[35] Slob E.A.W., Burgess S. (2020). A comparison of robust Mendelian randomization methods using summary data. Genet. Epidemiol..

[36] Greco M.F., Minelli C., Sheehan N.A., Thompson J.R. (2015). Detecting pleiotropy in Mendelian randomisation studies with summary data and a continuous outcome. Stat. Med..

[37] Burgess S., Bowden J., Fall T., Ingelsson E., Thompson S.G. (2017). Sensitivity Analyses for Robust Causal Inference from Mendelian Randomization Analyses with Multiple Genetic Variants. Epidemiology.

[38] Hemani G., Tilling K., Davey Smith G. (2017). Orienting the causal relationship between imprecisely measured traits using GWAS summary data. PLoS Genet..

[39] Huang J., Khademi M., Fugger L., Lindhe Ö., Novakova L., Axelsson M., Malmeström C., Constantinescu C., Lycke J., Piehl F. (2020). Inflammation-related plasma and CSF biomarkers for multiple sclerosis. Proc. Natl. Acad. Sci. USA.

[40] Ruiz F., Vigne S., Pot C. (2019). Resolution of inflammation during multiple sclerosis. Semin. Immunopathol..

[41] Pegoretti V., Swanson K.A., Bethea J.R., Probert L., Eisel U.L.M., Fischer R. (2020). Inflammation and Oxidative Stress in Multiple Sclerosis: Consequences for Therapy Development. Oxidative Med. Cell. Longev..

[42] Jank L., Bhargava P. (2024). Relationship Between Multiple Sclerosis, Gut Dysbiosis, and Inflammation: Considerations for Treatment. Neurol. Clin..

[43] Kuhlmann T., Ludwin S., Prat A., Antel J., Brück W., Lassmann H. (2017). An updated histological classification system for multiple sclerosis lesions. Acta Neuropathol..

[44] Popescu B.F., Lucchinetti C.F. (2012). Meningeal and cortical grey matter pathology in multiple sclerosis. BMC Neurol..

[45] Hedegaard C.J., Krakauer M., Bendtzen K., Lund H., Sellebjerg F., Nielsen C.H. (2008). T helper cell type 1 (Th1), Th2 and Th17 responses to myelin basic protein and disease activity in multiple sclerosis. Immunology.

[46] Fahmi R.M., Ramadan B.M., Salah H., Elsaid A.F., Shehta N. (2021). Neutrophil-lymphocyte ratio as a marker for disability and activity in multiple sclerosis. Mult. Scler. Relat. Disord..

[47] Meuth S.G., Simon O.J., Grimm A., Melzer N., Herrmann A.M., Spitzer P., Landgraf P., Wiendl H. (2008). CNS inflammation and neuronal degeneration is aggravated by impaired CD200-CD200R-mediated macrophage silencing. J. Neuroimmunol..

[48] Qiuping Z., Jei X., Youxin J., Wei J., Chun L., Jin W., Qun W., Yan L., Chunsong H., Mingzhen Y. (2004). CC chemokine ligand 25 enhances resistance to apoptosis in CD4+ T cells from patients with T-cell lineage acute and chronic lymphocytic leukemia by means of livin activation. Cancer Res..

[49] Wu X., Sun M., Yang Z., Lu C., Wang Q., Wang H., Deng C., Liu Y., Yang Y. (2021). The Roles of CCR9/CCL25 in Inflammation and Inflammation-Associated Diseases. Front. Cell Dev. Biol..

[50] Wu W., Doan N., Said J., Karunasiri D., Pullarkat S.T. (2014). Strong expression of chemokine receptor CCR9 in diffuse large B-cell lymphoma and follicular lymphoma strongly correlates with gastrointestinal involvement. Hum. Pathol..

[51] Kalindjian S.B., Kadnur S.V., Hewson C.A., Venkateshappa C., Juluri S., Kristam R., Kulkarni B., Mohammed Z., Saxena R., Viswanadhan V.N. (2016). A New Series of Orally Bioavailable Chemokine Receptor 9 (CCR9) Antagonists; Possible Agents for the Treatment of Inflammatory Bowel Disease. J. Med. Chem..

[52] Zhang Y., Han J., Wu M., Xu L., Wang Y., Yuan W., Hua F., Fan H., Dong F., Qu X. (2019). Toll-Like Receptor 4 Promotes Th17 Lymphocyte Infiltration Via CCL25/CCR9 in Pathogenesis of Experimental Autoimmune Encephalomyelitis. J. Neuroimmune Pharmacol..

[53] Braitch M., Constantinescu C.S. (2010). The role of osteopontin in experimental autoimmune encephalomyelitis (EAE) and multiple sclerosis (MS). Inflamm. Allergy Drug Targets.

[54] Tubo N.J., Wurbel M.A., Charvat T.T., Schall T.J., Walters M.J., Campbell J.J. (2012). A systemically-administered small molecule antagonist of CCR9 acts as a tissue-selective inhibitor of lymphocyte trafficking. PLoS ONE.

[55] Trivedi P.J., Schmidt C., Bruns T. (2016). Letter: The therapeutic potential of targeting CCL25/CCR9 in colonic inflammatory bowel disease-reading between the lines. Aliment. Pharmacol. Ther..

[56] Vazirinejad R., Ahmadi Z., Kazemi Arababadi M., Hassanshahi G., Kennedy D. (2014). The biological functions, structure and sources of CXCL10 and its outstanding part in the pathophysiology of multiple sclerosis. Neuroimmunomodulation.

[57] Sørensen T.L., Tani M., Jensen J., Pierce V., Lucchinetti C., Folcik V.A., Qin S., Rottman J., Sellebjerg F., Strieter R.M. (1999). Expression of specific chemokines and chemokine receptors in the central nervous system of multiple sclerosis patients. J. Clin. Investig..

[58] Simpson J.E., Newcombe J., Cuzner M.L., Woodroofe M.N. (2000). Expression of the interferon-gamma-inducible chemokines IP-10 and Mig and their receptor, CXCR3, in multiple sclerosis lesions. Neuropathol. Appl. Neurobiol..

[59] Balashov K.E., Rottman J.B., Weiner H.L., Hancock W.W. (1999). CCR5(+) and CXCR3(+) T cells are increased in multiple sclerosis and their ligands MIP-1alpha and IP-10 are expressed in demyelinating brain lesions. Proc. Natl. Acad. Sci. USA.

[60] Sørensen T.L., Sellebjerg F., Jensen C.V., Strieter R.M., Ransohoff R.M. (2001). Chemokines CXCL10 and CCL2: Differential involvement in intrathecal inflammation in multiple sclerosis. Eur. J. Neurol..

[61] Comini-Frota E.R., Teixeira A.L., Angelo J.P., Andrade M.V., Brum D.G., Kaimen-Maciel D.R., Foss N.T., Donadi E.A. (2011). Evaluation of serum levels of chemokines during interferon-β treatment in multiple sclerosis patients: A 1-year, observational cohort study. CNS Drugs.

[62] Yan Q., Elliott J., Snider W.D. (1992). Brain-derived neurotrophic factor rescues spinal motor neurons from axotomy-induced cell death. Nature.

[63] Mo L., Yang Z., Zhang A., Li X. (2010). The repair of the injured adult rat hippocampus with NT-3-chitosan carriers. Biomaterials.

[64] Rosenberg S.S., Ng B.K., Chan J.R. (2006). The quest for remyelination: A new role for neurotrophins and their receptors. Brain Pathol..

[65] McTigue D.M., Horner P.J., Stokes B.T., Gage F.H. (1998). Neurotrophin-3 and brain-derived neurotrophic factor induce oligodendrocyte proliferation and myelination of regenerating axons in the contused adult rat spinal cord. J. Neurosci..

[66] Baudet C., Pozas E., Adameyko I., Andersson E., Ericson J., Ernfors P. (2008). Retrograde signaling onto Ret during motor nerve terminal maturation. J. Neurosci..

